# Non-Apoptotic Cell Death Signaling Pathways in Melanoma

**DOI:** 10.3390/ijms21082980

**Published:** 2020-04-23

**Authors:** Mariusz L. Hartman

**Affiliations:** Department of Molecular Biology of Cancer, Medical University of Lodz, 6/8 Mazowiecka Street, 92-215 Lodz, Poland; mariusz.hartman@umed.lodz.pl; Tel.: +48-42-272-57-03

**Keywords:** autophagy, differentiation, drug resistance, ferroptosis, melanoma, necroptosis, parthanatos, pyroptosis, reactive oxygen species (ROS), targeted therapy

## Abstract

Resisting cell death is a hallmark of cancer. Disturbances in the execution of cell death programs promote carcinogenesis and survival of cancer cells under unfavorable conditions, including exposition to anti-cancer therapies. Specific modalities of regulated cell death (RCD) have been classified based on different criteria, including morphological features, biochemical alterations and immunological consequences. Although melanoma cells are broadly equipped with the anti-apoptotic machinery and recurrent genetic alterations in the components of the RAS/RAF/MEK/ERK signaling markedly contribute to the pro-survival phenotype of melanoma, the roles of autophagy-dependent cell death, necroptosis, ferroptosis, pyroptosis, and parthanatos have recently gained great interest. These signaling cascades are involved in melanoma cell response and resistance to the therapeutics used in the clinic, including inhibitors of BRAF^mut^ and MEK1/2, and immunotherapy. In addition, the relationships between sensitivity to non-apoptotic cell death routes and specific cell phenotypes have been demonstrated, suggesting that plasticity of melanoma cells can be exploited to modulate response of these cells to different cell death stimuli. In this review, the current knowledge on the non-apoptotic cell death signaling pathways in melanoma cell biology and response to anti-cancer drugs has been discussed.

## 1. Introduction

Cell death is an essential biological process that controls development and homeostasis in the organism [1,2]. Deregulation of cell death execution is a hallmark of cancer that promotes cancer development and progression, and is implicated in the resistance of cancer cells to therapies [3,4]. Regulated cell death (RCD) involves the activation of precisely controlled machinery that transmits signals promoting either cell survival or death. As RCD is executed by specific proteins, pharmacological targeting of these regulators can be exploited therapeutically [5,6,7,8]. For decades, apoptosis has been investigated as a major RCD modality involved in the survival of cancer cells and their response to anti-cancer drugs [9]. Apoptosis is initiated via one of two pathways: Extrinsic or intrinsic (mitochondrial). The extrinsic route of apoptosis couples the interactions between death receptors and death-promoting ligands, while intrinsic apoptosis is predominantly governed by the proteins from the B-cell leukemia/lymphoma 2 (BCL-2) family. This family consists of three groups of proteins: (i) Multi-domain pro-survival proteins such as BCL-2, myeloid cell leukemia 1 (MCL-1), B-cell lymphoma-extra large (BCL-X_L_), BCL-w, and BFL-1; (ii) multi-domain pro-apoptotic proteins BAX and BAK that execute apoptosis by compromising the outer mitochondrial membrane; (iii) a single-domain pro-apoptotic proteins, e.g., BCL-2-interacting mediator of cell death (BIM), that interact with either pro-survival or BAX/BAK proteins [7,8,10,11]. It is now evident that cell death signaling pathways are far more complex, and specific routes of cell death have been classified based on different criteria, including morphological features, biochemical alterations and immunological consequences (Table 1). However, the detailed molecular mechanisms of these modes of cell death are still poorly understood.

**Figure 1:**
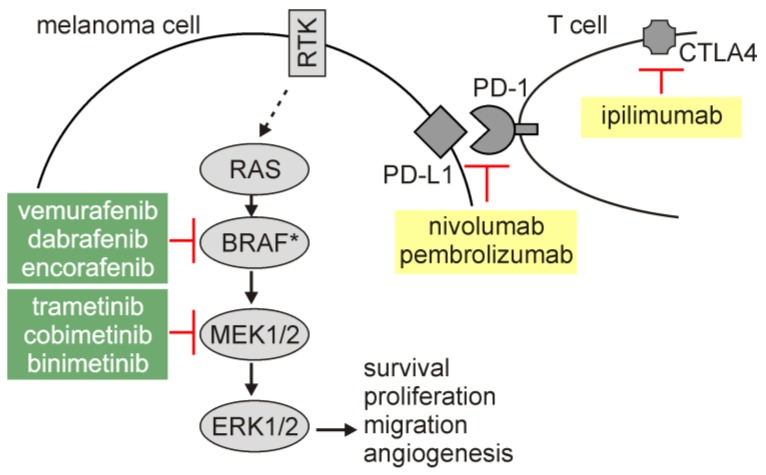
Targeted therapeutics and immunotherapy used in the treatment of melanoma patients. Melanoma cells exert hyperactivation of the RAS/RAF/MEK/ERK signaling pathway that regulates different cellular programs, including survival. Targeted therapeutics (shown in green background) inhibit activity of either mutated BRAF (BRAF*, V600E is the most frequent amino acid substitution) or MEK1/2. BRAFi and MEKi are used as a combinatory treatment regimen. Immunotherapy (shown in yellow background) includes checkpoint inhibitors: antibodies blocking either PD-1 (programmed death-1) or CTLA4 (cytotoxic T-lymphocyte associated protein 4). Both targets for immunotherapy are physiological inhibitors of T cell-mediated immune response. RTK, receptor tyrosine kinase.

Melanoma is the deadliest form of skin cancer with increasing incidence worldwide [16]. The hyperactivation of the RAS/RAF/MEK/ERK signaling pathway (hereafter MAPK), which is assessed in the vast majority of melanomas as a result of genetic alterations in *BRAF*, *RAS,* or *NF1* among others [17,18,19,20,21], contributes to the pro-survival phenotype of melanoma cells. A negative regulation of pro-apoptotic molecules (e.g., BIM) by oncogenic MAPK signaling has been reported [22], while anti-apoptotic proteins involved in the regulation of extrinsic and intrinsic apoptotic routes are largely overexpressed in melanoma [23,24]. Other signaling pathways [25], melanoma-specific transcriptional regulators [26] and post-transcriptional control [27] also extensively contribute to the capability of melanoma cells to counteract unfavorable conditions, including exposition to anti-cancer therapies. In addition, microenvironment-mediated regulation of expression of pro-survival molecules, including MCL-1, BCL-X_L_, and BFL-1 [28,29,30], supports a remarkable adaptive capabilities of melanoma cells. Despite a tremendous advances in the therapeutic options for melanoma patients (Figure 1), inability or limited vulnerability of melanoma cells to induction of apoptosis in response to inhibitors of BRAF^mut^ (BRAFi) and MEK (MEKi) [31,32,33,34,35,36,37,38], and escape from immunotherapy [39,40,41] are the reasons for re-growth of drug-resistant disease. In this respect, research on the mechanisms of the non-apoptotic cell death modalities is attractive in melanoma.

This review summarizes current knowledge on the role of non-apoptotic cell death signaling pathways in melanoma development and progression, as well as in response of melanoma cells to currently used therapeutics, i.e., BRAFi and MEKi, and immunotherapy.

## 2. Autophagy

### 2.1. An Overview of Autophagy and Autophagy-Dependent Cell Death

**Figure 2:**
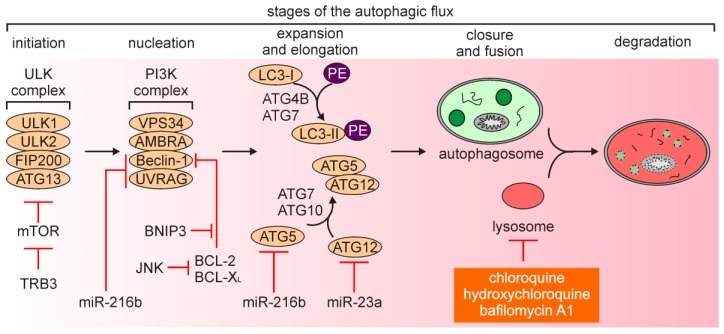
Stages and machinery of autophagy. Under nutrient starvation and other autophagy-inducing conditions, the autophagy flux is initiated by formation of the Unc51-like autophagy activating kinase (ULK) complex. The activated ULK complex participates in the recruitment of other proteins that constitute the phosphoinositide 3-kinase (PI3K) complex and proceed assembly of an initial double-membrane structure (phagophore). This stage of autophagy can be additionally regulated by the apoptosis-related proteins such as B-cell leukemia/lymphoma 2 (BCL-2) and B-cell lymphoma-extra large (BCL-X_L_) as they can sequester Beclin-1. Elongation is mediated to form autophagosomes by two ubiquitin-like systems: (1) Autophagy-related genes(ATG)5/ATG12 conjugation system, and (2) ATG4B- and ATG7-mediated processing and phosphatidylethanolamine (PE) conjugation to light chain 3 (LC3)-I (LC3-I:LC3-II conversion). PE-conjugated LC3-II associates with the autophagosome. The maturation of autophagosome is associated with the delivery of misfolded proteins and/or damaged organelles, and then autophagosome is fused with the lysosomes. The cargo is exposed to the lysosomal hydrolases, which are active under acidic conditions. Compounds that affect the autophagic flux by disturbing lysosome acidification are shown in orange background. Mechanisms of autophagy regulation that have been shown in melanoma are additionally included.

Autophagy is a catabolic process, in which proteins, bulk cytoplasm, and/or organelles are incorporated into double-membrane intracellular vesicles to be recycled within lysosomes. Thus, autophagy maintains cellular homeostasis by the removal of unfolded proteins and damaged organelles [42,43,44,45]. Autophagy can be executed either non-selectively (macroautophagy or autophagy) or in a selective manner to remove specific organelles, e.g., damaged mitochondria (mitophagy) [46] and peroxisomes (pexophagy) [47]. Autophagy is sustained at a low level in the majority of cells, while its efficiency can be affected by a number of stimuli [48]. Autophagy involves five stages: (1) Initiation, (2) nucleation of the double-membrane vesicles (phagophores, further extended to the autophagosomes), (3) expansion and elongation, (4) closure and fusion of the autophagosomes with the lysosomes, and (5) degradation of intravesicular content (Figure 2) [42,49]. Autophagy-related genes (*ATG*s) extensively contribute to different stages of autophagy execution. The formation of autophagosome is initiated by the protein complex composed of Unc51-like autophagy activating kinase 1 (ULK1) and ULK2 [50,51], a scaffolding protein 200 kDa focal adhesion kinase family-interacting protein (FIP200) [52], and ATG13 that serves as an adaptor protein [53]. The ULK complex transmits stress signals, such as nutrient or energy deprivation, to the autophagosome-forming sites as it regulates phosphorylation of downstream proteins. This protein complex is negatively regulated by the mechanistic target of rapamycin (mTOR) signaling pathway. The vesicle nucleation is proceeded by the phosphoinositide 3-kinase (PI3K) complex that includes VPS34/PIK3C3 and Beclin-1. Beclin-1, a BH3-only protein from BCL-2 family of proteins, is phosphorylated by ULK1 and acts as a scaffold facilitating localization of other autophagic proteins [54]. Beclin-1 is also a mechanistic link between autophagy and apoptosis as anti-apoptotic proteins BCL-2 and BCL-X_L_ can interact with Beclin-1 to interrupt both the Beclin-1/VPS34 complex and the interaction with ultraviolet radiation resistance associated gene (UVRAG) [55]. Phosphorylation of VPS34 restricts its interaction with Beclin-1, while activating molecule in Beclin-1-regulated autophagy (AMBRA) stabilizes the PI3K complex by binding Beclin-1. In addition, the hypoxia-responsive gene encodes BCL-2/adenovirus E1B 19 kDa protein-interacting protein 3 (BNIP3), which is contextually implicated in the regulation of apoptosis and autophagy [56]. BNIP3 can displace Beclin-1 from BCL-2 and activate autophagy (Figure 2) [57]. Nucleation is followed by the expansion and elongation of the autophagosome membrane, and this stage is regulated by microtubule-associated protein light chain 3 (LC3) protein. Processing and conjugation of LC3-I with phosphatidylethanolamine (PE) is catalyzed by ATG4B and ATG7, and leads to the formation of LC3B-II. While LC3-I remains in the cytosol, LC3-II is incorporated into both the outer and the inner membrane of the autophagosome, and therefore LC3-II is largely considered as a marker of autophagy induction [54]. Also, an adaptor protein sequestosome 1 (SQTM1, known as p62), which directs substrates to the autophagosomes, is degraded with its cargo proteins and can be used as a read-out of the autophagic flux [58,59]. Another conjugation system is under control of ATG7 and ATG10, and regulates the formation of the ATG5/ATG12 conjugate. Autophagosomes subsequently fuse with lysosomes that is facilitated by syntaxin 17 (STX17). This is followed by the formation of autophagolysosomes, in which the cargo is digested by hydrolases that demand low pH for their activity. This stage of autophagy can be inhibited by chloroquine, hydroxychloroquine and bafilomycin A1 that disturb acidification of the lysosomes (Figure 2) [55]. The role of autophagy still remains elusive as it can be either a cytoprotective response or followed by induction of different modalities of cell death [60,61,62]. According to the most current recommendations of nomenclature, the term “autophagy-dependent cell death” can be used exclusively when observed cell death is strictly reliant on the autophagic machinery [5,63].

### 2.2. Autophagy in Melanoma

In general, autophagy has been widely considered as a tumor suppressor mechanism at the stage of carcinogenesis, while tumor-promoting role of autophagy has been associated with progression of cancer, including melanoma [55,64,65,66]. It has been demonstrated that autophagy suppressed melanomagenesis by promoting senescence, and decreased expression of *ATG5* was sufficient to preclude this process [67]. In addition, exposure to ultraviolet A (UVA) upregulated p62/SQSTM1 and triggered p62-dependent response that involved nuclear factor erythroid 2-related factor 2 (NRF-2 encoded by *NFE2L2*) activity to counteract oxidative stress [68], while simulated sunlight irradiation was associated with induction of mitophagy [69]. In turn, low efficiency of autophagy execution promoted survival of melanoma cells exposed to UV irradiation [70]. On the contrary, others have demonstrated that loss of *ATG7* in a BRAF^V600E^/*PTEN*^null^ model of melanoma was associated with increased oxidative stress and senescence that prevented melanoma tumorigenesis [71], suggesting that inhibition of autophagy may also play a tumor-suppressing role. Notably, an oncogenic activation of the MAPK signaling pathway has been associated with the regulation of autophagy in melanoma [72,73]. BRAF^V600E^ has been shown to increase endoplasmic reticulum (ER) stress that was responsible for high basal autophagy in melanoma [74]. Mechanistically, c-JUN N-terminal kinase (JNK)-mediated phosphorylation of BCL-2 and BCL-X_L_ released Beclin-1, while Tribbles pseudokinase 3 (TRB3) additionally inhibited mTOR signaling (Figure 2) [74]. A hyperactive MAPK signaling pathway regulated cAMP-responsive element binding protein (CREB)-dependent expression of *NOXA*, which promoted autophagy in melanoma cells upon nutrient deprivation [75]. BRAF^V600E^-mediated starvation-induced autophagy was also shown in uveal melanoma [76].

Melanoma cells employ autophagy as an adaptive mechanism to the microenvironment insults, including acidic conditions [77], while inhibition of autophagy has been shown when low pH was accompanied with glucose deprivation [78] or combined glucose starvation and hypoxia [79]. Autophagy was also essential for survival of melanoma cells exposed to fluctuating oxygen pressure [80], suggesting an important role of autophagy during metastasis. In addition, induction of autophagy in melanoma cells can modulate tumor microenvironment. The comparison of secretome of autophagy^low^ and autophagy^high^ melanoma cells revealed that several proteins with established roles in inflammation and tumorigenesis, including interleukin-1β (IL-1β), chemokine C-X-C motif ligand 8 (CXCL8), leukemia inhibitory factor (LIF), family with sequence similarity 3 member C (FAM3), and dickkopf 3 (DKK3), were secreted at higher levels by melanoma cells with high activity of autophagy [81]. Notably, also patient-derived serum samples from melanoma patients with high level of autophagy flux have shown high levels of these proteins [81], suggesting that they can serve as surrogates for intra-tumoral autophagy dynamics. Elevated autophagy accompanying melanoma progression has been associated with loss of galectin-3 [82], increased levels of sirtuin 1 (SIRT1) [83], SIRT6 [84,85], BNIP3 [86], and long non-coding RNA ZNNT1 [87], and enhanced activity of signaling pathways such as AKT, independent of the mutational status of *BRAF* [88]. A heterozygous loss of *ATG5* enhanced melanoma metastasis and predicted poor overall patient survival [89]. In addition, miR-23a has been identified as a negative regulator of ATG12 (Figure 2), while ATG12 regulated melanoma cell invasion and migration through AMP-activated protein kinase-RAS homolog family member A (AMPK-RhoA) pathway [90]. Accordingly, expression of miR-23a was decreased in metastatic melanoma cell lines, and miR-23a level was significantly lower in serum of patients with metastatic melanoma [90]. An autophagy-independent role of p62/SQSTM1 has been ascribed to the control of melanoma metastasis by recruiting RNA-binding proteins in cooperation with insulin-like growth factor 2 mRNA-binding protein 1 (IGF2BP1) to stabilize transcripts of a number of pro-metastatic factors [91]. Notably, expression of several genes related to autophagy such as *SQSTM1*, *AMBRA1*, WD repeat domain phosphoinositide-interacting 1 (*WIPI-1*), peroxisomal biogenesis factor 3 (*PEX3*) and BCL-2-associating athanogene-1 (*BAG1*) has been indicated as prognostic in melanoma [92,93,94,95], and single nucleotide polymorphisms (SNPs) have been found in others [96]. Certain regulators relevant for melanoma can inhibit autophagy as shown for neural cell adhesion molecule (NCAM) [97], minichromosome maintenance protein 7 (MCM7) [98] and Wingless-type (WNT)/β-catenin cascade [99]. In line with this, high level of WNT5a, which regulates the activity of non-canonical WNT signaling pathway, and low level of β-catenin were associated with increased basal autophagy in melanoma cells [100]. In turn, downregulation of ATG5 in WNT5a^high^ melanoma cells was associated with lower level of WNT5a and increased level of β-catenin [100], supporting the conclusion that autophagy is inversely correlated with the activity of canonical WNT/β-catenin signaling pathway.

The impact of autophagy on immune response has also been investigated. In a mouse model of melanoma, T cell-mediated response was found equivalent in autophagy-competent mice and mice with the loss of autophagy-associated genes or pharmacological inhibition of autophagy by chloroquine [101]. It has also been demonstrated, however, that targeting Beclin-1 in melanoma cells increased the infiltration of natural killer (NK) cells into melanoma tumors that was dependent on increased expression of the C-C motif chemokine ligand 5 (*CCL5*). Induction of CCL5 was also reported upon inhibition of other autophagy proteins, including ATG5 and p62/SQSTM1, or pharmacological inhibition of autophagy. In addition, a positive correlation between CCL5 and attraction of NK cells was shown in melanoma patients, and a high expression of *CCL5* was correlated with improved patient survival [102,103]. This suggests that blocking autophagy may induce beneficial immune response, although it has been demonstrated that loss of BNIP3 significantly reduced phagocytic clearance of melanoma cells undergoing cell death [104].

In particular, autophagy is used by melanoma cells to counteract drug activity and drug-induced changes in tumor microenvironment, thus autophagy correlates with clinical outcome and largely contributes to resistance of melanoma cells to therapeutics [105,106,107,108]. Autophagy assessed in tumor specimens obtained from melanoma patients enrolled in a phase II trial of temozolomide and sorafenib has revealed that patients whose tumors displayed high autophagy activity had a worse clinical outcome [109]. Increased level of BNIP3 accompanying activation of autophagy was correlated with poor efficacy of pembrolizumab in melanoma patients [110]. In addition, patients with BRAF-mutant melanoma that exhibited high level of autophagy were characterized with poorer response to BRAFi [111]. It has been shown that BRAFi and combined BRAFi and MEKi upregulated autophagy as a cytoprotective mechanism [111]. Autophagy accompanied melanoma cell senescence induced by encorafenib [37,38], while loss of LC3 protein substantially prevented from encorafenib-induced senescence [38]. It has also been demonstrated that treatment with dabrafenib more markedly inhibited growth and induced senescence in tumors derived from *ATG7*-deficient melanoma cells [71], further indicating that efficient autophagic flux limits the response of melanoma cells to anti-cancer drugs. Autophagy was also assessed at a higher level in BRAFi-resistant melanomas, and significant tumor regression was reported when drug-resistant tumors were treated with a combination of BRAF^mut^ and autophagy inhibitors [111]. Others have shown, however, that either pharmacological inhibition or *ATG5* knockdown was insufficient to re-sensitize vemurafenib-resistant melanoma cells to this BRAFi, although higher activity of autophagy was assessed in resistant cells compared with drug-naïve counterparts [112]. As MEK-mediated resistance to vemurafenib was found in these cells, a combination of MEKi and autophagy inhibitor efficiently increased cell death [112], suggesting that specific mechanisms of resistance may affect melanoma cell dependence on autophagy.

Several mechanisms on drug-induced autophagy have been reported. It has been shown that vemurafenib reduced miR-216b level that resulted in upregulation of Beclin-1, ATG5 and UVRAG [113]. Dabrafenib induced autophagy by downregulation of miR-26a following upregulation of HMGB1 in melanoma [114]. In addition, it has been shown that combined vemurafenib and trametinib treatment induced SEC61-dependent translocation of components of the MAPK signaling pathway to endoplasmic reticulum [115]. Following this translocation, ERK was reactivated by protein kinase RNA-like endoplasmic reticulum kinase (PERK)-mediated phosphorylation. Re-phosphorylated ERK induced activating transcription factor 4 (ATF4) to trigger cytoprotective autophagy [115]. Moreover, it has been shown that vemurafenib-resistant melanoma cells employed autophagosomes to secret ATP and enhance cell migration and invasion [116]. Compromising autophagy by downregulation of several autophagy-related genes significantly reduced secretion of ATP and, consequently, cell invasion [116]. Inhibition of AMPK-α1 in vemurafenib-resistant melanoma cells impaired autophagy that was associated with decreased formation of the autophagosomes [117]. Drug resistance of melanoma cells can be additionally supported by elevated levels of other autophagy-related proteins, including BAG3 [118]. Available data suggest, however, that not all melanoma patients may benefit from inhibition of autophagy. Mechanistic study on induction of autophagy upon inhibition of BRAF^V600E^ has shown that BRAFi disrupted ERK-mediated phosphorylation of transcription factor EB (TFEB), allowing its nuclear translocation and activation of a cellular program that controls lysosome biogenesis [119]. This was, surprisingly, followed by tumor suppression and decreased metastasis in a mouse model of melanoma [119]. On the other hand, suppression of Yin Yang 1 (YY1), which is a cofactor of TFEB and contributes to the regulation of genes related to autophagy and lysosome biogenesis, evoked enhanced anti-melanoma efficiency of vemurafenib both in vitro and in vivo [120]. Interestingly, another transcription factor that is related to TFEB, microphthalmia-associated transcription factor (MITF) has been shown to bind to the promoters of genes encoding lysosomal and autophagosomal proteins in melanoma. MITF-regulated genes were, however, distinct from these activated by TFEB and TFE3 [121]. Modulating the level of MITF largely affected the response of melanoma cells to starvation-induced autophagy [121]. Considering an important role of MITF in the regulation of melanoma cell phenotype and response to targeted drugs, including diverse execution of MITF-dependent programs in drug-resistant melanoma cells [122,123,124], further research is necessary to delineate a relationship between activity of MITF and TFE transcription factors and consequences of manipulation of autophagy-lysosomal signaling in melanoma cells.

## 3. Necroptosis

### 3.1. An Overview of Necroptotic Signaling Pathway

**Figure 3:**
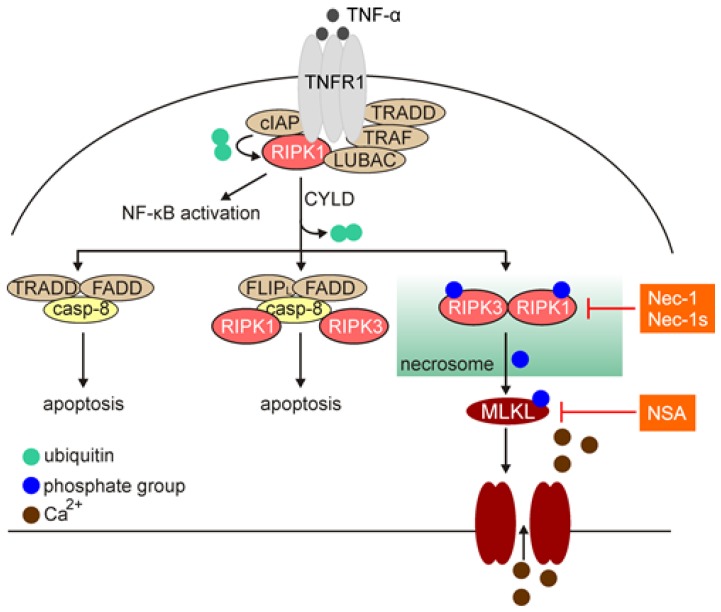
Necroptotic signaling pathway exemplified by tumor necrosis factor alpha (TNF-α)-mediated activation of tumor necrosis factor receptor (TNFR)1. Activated TNFR1 forms a multi-protein complex I that transmit either pro-survival response (NF-κB activation) or cell death response, which is mediated by cylindromatosis (CYLD)-dependent deubiquitylation of receptor-interacting protein kinase 1 (RIPK1). Complexes-IIa/b are formed when the activation of caspase-8 can proceed apoptosis. Upon inhibition of caspase-8, complex IIc (necrosome) mediates activation of mixed lineage kinase domain-like (MLKL) by phosphorylation at Ser^358^ that results in pore formation in the plasma membrane and Ca^2+^ influx. Compounds that inhibit necroptosis, including necrostatin-1 (Nec-1), necrostatin-1s (Nec-1s), and necrosulfonamide (NSA) are shown in orange background.

Necroptosis has been first described in 2005 as a caspase-independent cell death modality induced by perturbations of extra- or intra-cellular homeostasis when the apoptotic machinery is inhibited [125]. Necroptosis shares morphological features with necrosis, including membrane permeabilization, cell swelling and loss of cell integrity (Table 1), and in part, structural organization with extrinsic pathway of apoptosis (Figure 3) [126]. Necroptosis is associated with a substantial production of reactive oxygen species (ROS), hyperactivation of poly(ADP-ribose) polymerase 1 (PARP1) and depletion of ATP [127,128]. The cell rupture accompanying necroptotic cell death leads to the release of damage-associated molecular patterns (DAMPs) and cytokines, including IL-1α, IL-1β, high mobility group box 1 (HMGB1) and IL-33, thus triggers robust inflammatory response (Table 1). Necroptosis is largely reliant on sequential activation of receptor-interacting protein kinase 1 (RIPK1), RIPK3 and mixed lineage kinase domain-like (MLKL) [5], and necrostatin-1 (Nec-1) and necrostatin-1s (Nec-1s), which selectively stabilize an inactive conformation of RIPK1, inhibit the necroptotic cell death pathway [129,130]. Mechanistically, necroptosis is activated by a plethora of different ligands, including tumor necrosis factor alpha (TNF-α), FAS ligand (FASL), TNF-related apoptosis-inducing ligand (TRAIL), interferon gamma (IFN-γ) and lipopolysaccharide (LPS) that engage members of the tumor necrosis factor receptor (TNFR) family, FAS, T cell receptors (TCRs), and pattern recognition receptors (PRRs) [126,131]. TNF-α-mediated necroptosis has been largely investigated so far (Figure 3). Following binding TNF-α, trimers of TNFR1 recruit multiple proteins to form membrane-bound complex I. This complex includes TNFR-associated death domain (TRADD), RIPK1, cellular inhibitor of apoptosis protein 1 and 2 (cIAP1 and 2), TNFR-associated factor 2 and 5 (TRAF2 and 5), and linear ubiquitin chain assembly complex (LUBAC), in addition to TNFR [132]. RIPK1 is a member of serine-threonine kinase family, and consists of three major domains: (i) C-terminal domain that interacts with death domain receptors (DRs) and/or adaptor protein TRADD; (ii) central region that is responsible for nuclear factor kappa B (NF-κB) activation and includes receptor-interacting protein homotypic interaction motif (RHIM) responsible for the interaction with RIPK3; and (iii) a regulatory N-terminal fragment (T-loop) that contains Asp-Leu-Gly residues [133]. Within complex I, cIAPs can ubiquitinate RIPK1 and induce the pro-survival NF-κB signaling pathway. On the other hand, ligand-bound TNFR1 can be internalized, and RIPK1 is deubiquitinated by cylindromatosis (CYLD) that switches the pathway towards activation of cell death. This is associated with the formation of a cytoplasmic multimeric complex comprising RIPK1, TRADD, caspase-8, and FAS-associated death domain protein (FADD), which is known as complex II. Complex II links the activation of apoptotic (complex IIa and IIb) and necroptotic (complex IIc; necrosome) pathways. Activated caspase-8 cleaves and inactivates RIPK1 at Asp^324^, RIPK3, and CYLD, and leads to the execution of apoptosis, while necroptosis is proceeded upon inhibition of caspase-8 [131,133]. This is followed by an activatory autophosphorylation of RIPK1 at its N-terminal Ser^161^. Activated RIPK1 interacts with RIPK3, and subsequently RIPK3 phosphorylates MLKL. MLKL is recruited to the plasma membrane, where it interacts with phospholipids to enhance membrane permeability and execute necroptosis (Figure 3) [126,131]. The contribution of heat shock protein 90 (HSP90) to MLKL oligomerization and translocation has been ascribed [134,135]. Loss of plasma membrane integrity has been associated with the influx of calcium ions and exposure of phosphatidylserine, and both processes are executed in an MLKL-dependent manner [136]. This is also associated with ESCRT-II-dependent generation of the plasma membrane “bubbles”, which are however, dissimilar to the apoptotic bodies as they do not contain cytosolic remnants [136]. In addition, phosphorylated MLKL can be released within exosomes that has been suggested as a mechanism for cell self-restricting activity of MLKL in the execution of necroptosis [137]. It has also been demonstrated that necroptosis can be induced in certain cells independently of RIPK1, but still reliant on RIPK3 and MLKL [138], suggesting that activation of RIPK3 and MLKL is more universal biomarkers of necroptosis.

### 3.2. Necroptosis in Melanoma

Several studies have indicated that inducing necroptosis in melanoma may be limited as it was found that both *CYLD* [139] and *RIPK3* were poorly expressed in melanoma cell lines [140,141], although melanocytes and benign nevi expressed *RIPK3* at a high level [140]. This suggests that low expression of essential regulators of the necroptotic pathway may be an intrinsic feature of melanoma cells to evade necroptosis. It has also been reported that low level of RIPK1 in melanoma cells promoted induction of apoptosis [142], and RIPK1 regulated autophagy in melanoma cells upon ER stress [143]. The transforming growth factor β-activated kinase 1 (TAK1) could prevent from cell death by suppressing RIPK1 activity even if RIPK3 and MLKL were intrinsically expressed [144]. TAK1 inhibition sensitized melanoma cells to apoptosis induced by a combination of TNF-α and TRAIL [144], indicating that TAK1 regulated apoptosis-related role of RIPK1 in melanoma. In addition, inhibition of RIPK1 and loss of RIPK3 did not reduce lung metastasis in a mouse model of melanoma [145]. In another study, however, pre-treatment of mice with necrostatin-1 or a novel RIPK1 inhibitor, PK68, significantly suppressed lung metastasis by melanoma cells [146], suggesting that further research is needed to clarify the role of RIPK1 in melanoma progression.

In contrast to vemurafenib, it has been shown that dabrafenib inhibited RIPK3 activity in an ATP-competitive manner, and irrespectively of inhibition of BRAF^mut^ [140,147]. Dabrafenib disrupted RIPK3 and MLKL interactions that was associated with decreased levels of MLKL phosphorylated at Ser^358^ [147], and prevented from induction of necroptosis in RIPK3-expressing melanoma cells [140]. It has been demonstrated that dabrafenib could also protect normal cells from necroptosis-inducing compounds as evidenced for hepatocytes [147] and neurons [148]. These observations suggest that loss of necroptotic machinery in the majority of melanomas and inhibition of RIPK3 activity by drugs used in the clinic contribute to protection of melanoma cells from necroptosis. This also justifies the successful transition to dabrafenib administration in patients with toxic epidermal necrolysis induced after treatment with vemurafenib [149], and indicates that use of specific BRAF^mut^ inhibitor can be important when overall toxicity of the therapy is considered.

Strategies that allow to manipulate and reactivate the necroptosis-related machinery can be still relevant for triggering this type of cell death in melanoma cells. It has been shown that RIPK3-expressing melanoma cells were sensitive to necroptosis-inducing agents and cell death could be efficiently inhibited by necrostatin-1 [141], although gaining competence to trigger necroptosis upon overexpression of RIPK3 has been questioned by others [144]. Reconstitution of RIPK3 was sufficient to phosphorylate MLKL and induce necroptosis in response to FASL/IAP antagonist [140]. In addition, a pan-caspase inhibitor combined with radiotherapy, dacarbazine and hyperthermia significantly reduced melanoma growth by inducing necroptosis and increasing dendritic cell (DC) and CD8+ T-cell infiltration in the tumor microenvironment [150]. Necroptosis was induced in response of melanoma cells to photothermal therapy, but only within a narrow range of temperature [151]. Necroptosis was also reported as a way of dying in response of BRAF^V600E^ melanoma cells to inhibition of the mitochondrial complex I, and this was associated with augmented production of ROS [152]. In addition, combined re-establishment of p19^Arf^ and IFN-β signaling pathways was associated with induction of necroptosis in melanoma, and activation of immune response involving NK cells, T cells and neutrophils [153]. An interesting study published more recently has evidenced that intra-tumor delivery of MLKL-encoding mRNA elicited a potent anti-tumor immunity, even if tumors were defective for proteins that acted upstream of MLKL. Mechanistically, MLKL mRNA-induced T cell response required migration of DCs and interferon signaling. This strategy was effective at inhibiting both primary tumor growth and metastasis in a mouse model of melanoma, while it also complemented anti-melanoma activity of anti-PD1 immunotherapy [154]. Both UVB and UVC have also been shown to increase MLKL protein level [70], and vapor nanobubble photoporation-mediated delivery of MLKL protein to melanoma cells effectively induced necroptosis [155].

## 4. Ferroptosis

### 4.1. An Overview of Ferroptosis

**Figure 4:**
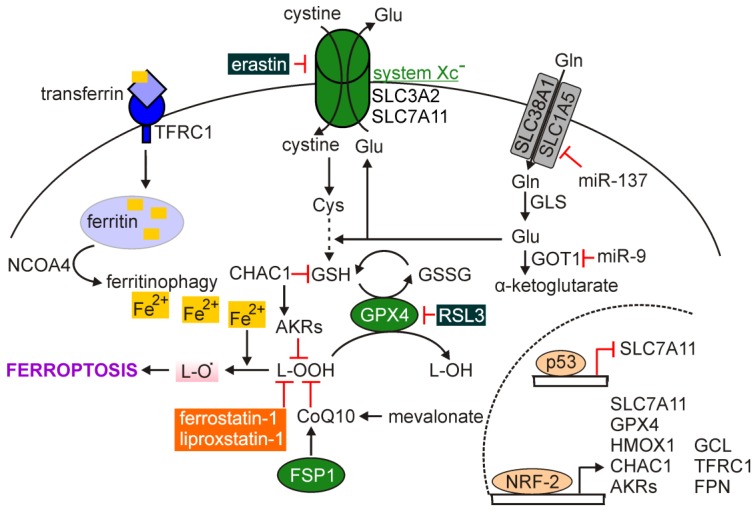
Regulation of ferroptosis. Transmembrane system X_C_^¯^ composed of SLC3A2 and SLC7A11 subunits sustains the glutathione (GSH) homeostasis. GPX4 catalyzes the glutathione-dependent reduction of lipid hydroperoxides (L-OOH) to lipid alcohols (L-OH) that prevents from the formation of highly reactive lipid alkoxy radicals (L-O^•^) and induction of ferroptosis. This process can also be prevented by the activity of FSP1, while Fe^2+^ released from ferritin in NCOA4-mediated ferritinophagy accelerates ferroptosis. Several genes encoding ferroptosis-regulating proteins are under transcriptional control of p53 and nuclear factor erythroid 2-related factor 2 (NRF-2). Compounds that inhibit ferroptosis are shown in orange background, and activators of ferroptosis are shown in dark green background. Mechanisms of ferroptosis regulation that have been demonstrated in melanoma are additionally included.

Ferroptosis is a regulated caspase-independent cell death modality characterized with the overwhelming production of ROS and accumulation of iron-dependent lipid peroxides [156,157,158]. Cells that undergo ferroptosis exhibit a typical necrotic morphology and dysmorphic mitochondrial phenotype, including shrunken mitochondria, increased mitochondrial membrane density and reduced level of intracellular NADH, but not ATP (Table 1) [159,160]. Several metabolic signaling pathways and related regulators have been shown to modulate cell sensitivity to ferroptosis in different experimental settings [159,161,162,163]. The immunomodulatory role of ferroptosis has also been investigated and discussed [164]. A member of the BCL-2 family, BH3-interacting domain death agonist (BID), which is truncated (tBID) during extrinsic apoptosis has been required for ferroptosis in neurons [165], suggesting an interconnection between ferroptotic and apoptotic cell death signaling pathways. More universally, ferroptosis is triggered in response to inhibition of glutathione (GSH) biosynthesis or inhibition of selenoprotein glutathione peroxidase 4 (GPX4) (Figure 4). It has also been reported that both transferrin (an iron-carrier protein) and glutaminolysis control ferroptosis that is triggered by deprivation of amino acids or cystine alone [161]. More recently, it has been demonstrated that cell death through ferroptosis can be antagonized by glutathione-independent ferroptosis suppressor protein 1 (FSP1) known also as apoptosis-inducing factor mitochondrial 2 (AIFM2), which acts in parallel to GPX4 [166,167]. It has been shown that FSP1 is recruited to the plasma membrane upon myristoylation to reduce coenzyme Q10 (CoQ10), which functions as a lipophilic antioxidant and prevents from the propagation of lipid peroxides [166]. Inhibition of FSP1 substantially synergized with inhibitors of GPX4 in ferroptosis induction in cancer cells [167]. Fine-tuned iron homeostasis interconnects autophagy and ferroptosis as selective degradation of ferritin, referred to as ferritinophagy, potentiates cell sensitivity to ferroptosis. Ferritinophagy promotes the increase of content of free intracellular iron, which is known as labile iron portion (LIP) [161,168,169], in a ROS-dependent manner [170]. Nuclear receptor coactivator 4 (NCOA4) mediates ferroptinophagy, and inhibition of autophagy or knockdown of NCOA4 prevented from the accumulation of LIP and ROS [171].

The disturbances in the synthesis of glutathione can be associated with inhibition of the cystine/glutamate antiporter named system X_C_^−^, which is composed of the twelve-pass transmembrane protein solute carrier family 7 member 11 (SLC7A11; xCT, light chain) connected to a single-pass transmembrane regulatory protein SLC3A2 (4F2, heavy chain) [13]. Inside the cell, reduction of cystine to cysteine occurs providing a substrate for glutathione synthesis and maintenance of intracellular redox homeostasis. Reduced glutathione is an essential intracellular antioxidant synthesized from amino acids: glycine, glutamate and cysteine in an ATP-dependent process involving cytosolic enzymes: Glutamate-cysteine ligase (GCL) and glutathione synthetase (GSS), while cysteine availability is a rate-limiting step in GSH synthesis [172]. In turn, GPX4 has been recognized as the main endogenous negative regulator of ferroptosis that acts by catalyzing the glutathione-dependent reduction of lipid hydroperoxides (L-OOH) to lipid alcohols (L-OH) (Figure 4) [173]. This prevents from the formation of markedly reactive lipid alkoxy radicals (L-O^•^) from lipid hydroperoxides [174]. When phospholipid hydroperoxides are not efficiently quenched by GPX4, they can trigger a reaction, which involves the transition metals such as iron (Fe(II)) [175]. Peroxidation of lipids can occur through autoxidation or enzyme activity, especially lipoxygenases (LOXs) [176]. It has been demonstrated that ferroptosis is induced upon preferential oxidation of polyunsaturated fatty acids (PUFAs) containing phosphatidylethanolamine, including arachidonic acid and adrenic acid [13]. Peroxidation of lipids reduces their ability to form fully functional cellular membranes that leads to the loss of membrane integrity. Accordingly, inhibition of acyl-CoA synthetase long chain family member 4 (ACSL4) and lysophosphatidylcholine acyltransferase 3 (LPCAT3), which contribute to the insertion and remodeling of long PUFAs into membrane phospholipids, prevented from induction of ferroptosis [13]. Several activators of ferroptosis have been identified, and this class of agents includes inhibitors of GPX4 (e.g., (1*S*,3*R*)-RSL3) and inhibitors of system X_C_^−^ (e.g., erastin) among others [173].

The majority of the proteins that regulate ferroptosis are under transcriptional control of NRF-2. NRF-2 regulates expression of *SLC7A11*, *GPX4,* and *GCL*, as well as genes related to iron metabolism, including transferrin receptor 1 (*TFRC1*) ferroportin (FPN encoded by *SLC40A1*) heme oxygenase 1 (*HMOX1*) and ferritin (*FTL*) [177,178]. Thus, NRF-2 modulates both basal redox homeostasis as NRF-2 is negatively regulated by Kelch-like ECH-associated protein 1 (KEAP-1), and antioxidant response during an excessive oxidative stress when KEAP1 is dissociated from NRF-2 and degraded [179].

### 4.2. Ferroptosis in Melanoma

Cancer cells are largely susceptible to perturbations of thiol metabolism and an excess of iron supply [180]. In melanoma, withstanding oxidative stress accompanying transient metabolic alterations has been recognized as a determinant of successful metastasis [181], suggesting that sensitivity of melanoma cells to ferroptosis may vary during melanoma development and progression. It has also been suggested that ferroptosis may be triggered upon DNA damage in melanoma cells [182]. In addition, several oncogenic pathways relevant for melanoma have been shown to render cancer cells susceptible to ferroptosis via the modulation of essential regulators of this type of cell death.

Initially, ferroptosis was postulated to be relevant exclusively in *RAS* mutant cancer cells, but it is now evident that this modality of cell death can be induced irrespectively of the mutational status of *RAS* [183,184,185]. Indeed, it has been demonstrated that mutated BRAF negatively regulated oxidative metabolism in melanoma cells [186], while BRAF inhibitors enhanced dependence of melanoma cells on the oxidative phosphorylation (OXPHOS) [186,187]. It has been hypothesized that chemosensitivity of OXPHOS^high^ cancer cells could rely on ROS accumulation and might be potentially executed by induction of ferroptosis [188]. This suggests that a metabolic switch induced by BRAFi can sensitize melanoma cells to ferroptosis-inducing agents, and SCL7A11 has been suggested as a marker of the susceptibility of metastatic melanoma cells to ferroptosis [189]. In line with this, acute treatment with vemurafenib or trametinib reduced transcript level of *SLC7A11* in BRAF^V600E^ melanoma cells [190]. Others have identified changes of the lipid metabolism, such as accumulation of PUFAs, in melanoma cells exposed to vemurafenib [191]. BAY 87-2243, an inhibitor that impairs OXPHOS by targeting the mitochondrial complex I, cooperated with vemurafenib in attenuation of melanoma tumor growth in vivo [187], although this could be related to induction of both ferroptosis and necroptosis as reported by others [152]. The metabolic switch of melanoma cells towards OXPHOS can be further extended by more complex phenotypic alterations accompanying acquired resistance to BRAFi and MEKi. It has been found that the acquisition of resistance was associated with an elevated dependence on the glutamine metabolism as BRAFi-resistant melanoma cells promoted glucose-derived glutamate synthesis and increase in glutathione content. In addition, drug-resistant cells exhibited a substantial activation of NRF-2 that was associated with activation of the pentose phosphate pathway involved in the regeneration of GSH, as well as increased expression of a component of system X_C_^−^ [192]. In another study, however, basal levels of GSH were significantly lower in vemurafenib-resistant cell lines than in matched drug-naïve cells [193]. It has been further demonstrated for different types of cancer, including melanoma, that persisting drug-resistant cells that exhibited a gene signature of mesenchymal-like state were more susceptible to inhibition of GPX4 [194]. A non-genetic addiction to GPX4 accompanied drug-resistant patient-derived melanoma cells reliant on transforming growth factor beta (TGF-β) [195]. In addition, *GPX4* knockout was lethal in chemoresistant, but not drug-naive, A375 melanoma cells, while this could be prevented by chemical inhibitors of ferroptosis such as ferrostatin-1. Ferrostatin-1 promoted tumor growth of A375^GPX4-/-^ cells xenografted into mice, which were treated with a combination of dabrafenib and trametinib, and withdrawal of ferrostatin-1 was associated with inhibition of the growth of GPX4^-/-^ tumors [196]. Furthermore, drug-resistant melanoma cells can dedifferentiate to different extent that is associated with loss of melanoma-specific transcription factor, MITF [123,193], and several studies have suggested that the dedifferentiation status can dictate sensitivity of cancer cells to ferroptosis. A large pharmacogenomics integration approach confirmed an inverse association between differentiation state of melanoma and its vulnerability to ferroptosis. BRAFi resistance-associated dedifferentiation increased the sensitivity to ferroptosis inducers, erastin and RSL3. In addition, levels of GSH were significantly correlated with the stage of cell dedifferentiation [193]. This suggests that glutathione can be a metabolic link between drug-induced dedifferentiation and sensitivity to ferroptosis-inducing agents, and this was confirmed by supplementation of the culture medium with GSH that rescued melanoma from ferroptosis [193]. Recently, cerebellar degeneration-related 1 antisense (CDR1as) has been identified as a novel marker of melanoma cell differentiation state, and depletion of CDR1as was associated with the metastatic potential of melanoma [197]. It has been found that CDR1as^high^ cells were substantially more sensitive to inhibitors of GPX4, thus induction of ferroptosis. In addition, high levels of CDR1as have been preferentially associated with melanoma cell state marked by low MITF level and high AXL level [197]. Thus, expression of CDR1as may serve as a determinant of phenotypic state of melanoma cells that may be targeted by inducers of ferroptosis.

Ferroptosis is also relevant in melanoma cell response to immunotherapy. Reflecting the inflammatory microenvironment by supplementation of IFN-γ or TNF-α to melanoma cell culture has provoked cell dedifferentiation, and was associated with increased susceptibility to ferroptosis induction [193]. It has been further demonstrated that CD8+ T cells activated by immunotherapy enhance lipid peroxidation in tumor cells, including melanoma. This contributed to anti-melanoma effect of immunotherapy as efficacy of anti-CTLA4 and anti-PD-1 antibodies was efficiently inhibited by liproxstatin-1, an inhibitor of ferroptosis. This indicated that immunotherapy-activated T cells can employ ferroptosis as a cytotoxic mechanism in melanoma cells [198]. Mechanistically, T cell-derived IFN-γ reduced expression of *SLC3A2* and *SLC7A11*, thus impairing the cystine uptake and affecting intracellular GSH supply. Analysis of transcriptomes of melanoma patients before and during nivolumab treatment revealed that clinical benefit was associated with reduced expression of *SLC3A2*, enhanced CD8+ T cell signature and expression of *IFNG* that was associated with improved overall patient survival [198].

The role of other regulators of ferroptosis have been investigated in melanoma. p53, encoded by the *TP53*, is frequently mutated in cancer, but very infrequent loss-of-function genetic alterations of *TP53* are found in melanoma [17], suggesting that activity of p53 may be exploited to induce ferroptosis. The tumor suppressor activity of p53 has been linked to inhibition of cell cycle, as well as induction of senescence and apoptosis, while the contribution of p53 to the control of cellular redox state and metabolism may be associated with the regulation of ferroptosis by this transcription factor [199,200]. It has been postulated that two divergent p53 functions can be exhibited depending on the stimuli. When DNA damage occurs, p53 functions as a regulator of cell cycle arrest and apoptosis, whereas accumulation of ROS beyond the state of reversal implicates p53 in cell response to this insult [201]. This suggests that p53 might act as a rheostat that prevents from ferroptosis under low oxidative stress, whereas it promotes ferroptosis under high ROS stress. This has been supported by reports showing that p53 repressed *SLC7A11* and eventually limited cystine uptake (Figure 4) [202]. The contribution of p53 to the regulation of expression of several other genes encoding proteins involved in redox homeostasis has been established [201]. Interestingly, it has also been shown that p53 activated expression of nerve growth factor receptor (NGFR, also known as CD271), and negative feedback was driven by NGFR to inactivate p53 by both promoting mouse double minute 2 (MDM2)-dependent proteolysis of p53 and inhibiting interaction between p53 and DNA [203]. As enhanced NGFR/CD271 level is associated with the neural crest-like state of melanoma cells [193], and vemurafenib- or trametinib-resistant cell lines frequently exhibit higher percentages of NGFR^high^ cells compared with matched drug-naïve cell lines [124], further research is necessary to delineate whether and how p53 contributes to increased sensitivity of drug-resistant melanoma cells to ferroptosis.

Also the role of NRF-2 has been investigated in melanoma (Figure 4). Enhanced activity of NRF-2 in melanoma was connected to genetic alterations in *KEAP1*, and was responsible for intrinsic resistance of melanoma cells to cisplatin and dacarbazine [204]. In a recent study, the role of NRF-2 has been more extensively investigated in melanoma cells resistant to ferroptosis [189]. It has been shown that ferroptosis-resistant cells upregulated NRF-2, which induced expression of an early ferroptotic marker glutathione-specific gamma-glutamylcyclotransferase 1 (CHAC1), and the aldo-keto reductases (AKRs) such as AKR1C1, AKR1C2, and AKR1C3 to degrade the lipid peroxides and prevent from ferroptotic cell death. Inhibition of AKR activity sensitized melanoma cells to ferroptosis confirming the role of these enzymes in prevention of ferroptosis induction [189]. In addition, NRF-2 has been implicated in UV-induced expression of PD-L1 in melanoma. It has been shown that depletion of NRF-2 was associated with tumor infiltration by CD8+ and CD4+ T cells, whereas combined inhibition of NRF-2 and PD-1 demonstrated enhanced anti-melanoma effect [205]. This suggests that NRF-2 may also be considered as an alternative target to inhibit the PD-1/PD-L1 signaling. Others reported that both tumor growth and lung metastasis of melanoma were enhanced in *Nrf2*-null mice [206], and reduced mRNA level of NRF-2 was observed during melanomagenesis [207].

The role of microRNAs in the regulation of ferroptosis has also been demonstrated in melanoma (Figure 4). miR-137 negatively regulated ferroptosis, which was shown both in vitro and in vivo, as it directly inhibited glutamine transporter SLC1A5 in melanoma cells [208]. It has been demonstrated that ectopic expression of miR-137 suppressed SLC1A5 that was associated with reduced uptake of glutamine and accumulation of malondialdehyde [208]. Furthermore, it has been shown that miR-9 inhibited ferroptosis by targeting glutamicoxaloacetic transaminase 1 (GOT1), which mediates the conversion of glutamate to α-ketoglutarate [209]. Inhibition of miR-9 led to lipid peroxidation and iron accumulation [209].

## 5. Pyroptosis

### 5.1. An Overview of Pyroptosis

Pyroptosis is a type of a lytic cell death associated with the damage of cellular membrane by members of the gasdermin protein family, and it is usually triggered by the activation of inflammation-related caspases (Table 1) [5]. The term pyroptosis was originally coined to define a modality of RCD dependent on inflammatory caspase-1, and the role of pyroptosis was assigned to innate immunity as a way of clearance of bacterial and viral infections [210]. Growing evidence, however, has indicated that pyroptosis can also be induced in cancer cells in response to chemotherapy [211]. Pyroptosis is usually executed by two proteins gasdermin D (GSDMD) and gasdermin E (GSDME), which is encoded by *DFNA5*. Other members of the gasdermin family of proteins include GSDMA, GSDMB, GSDMC, and DFNB59, and they all exhibit membrane perforation activity, which is associated with induction of pyroptosis. Gasdermin proteins form pores, which have a diameter of 10–14 nm and contain sixteen protomers [15,212].

**Figure 5:**
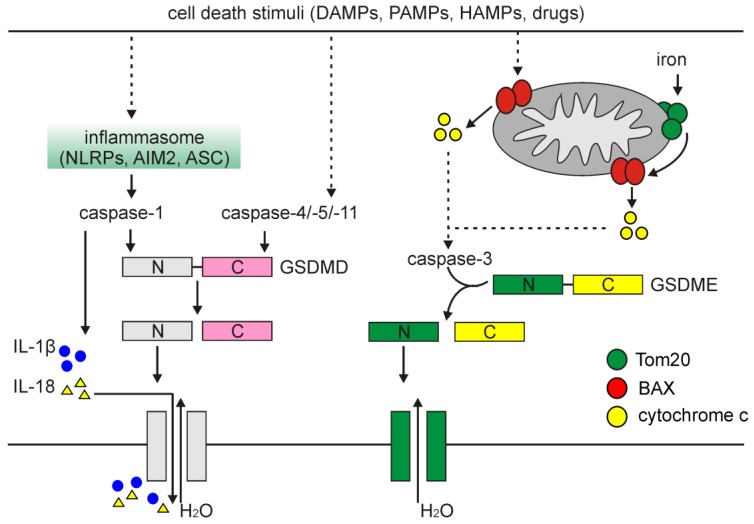
Signaling pathways that regulate pyroptosis. Pyroptosis-inducing stimuli activate inflammatory caspases, and this is followed by (i) processing of gasdermin D (GSDMD) by cleavage of the hinge region between C- and N-terminal domains to generate fragments that form pores in the plasma membrane, and (ii) processing of inflammatory cytokines, e.g., IL-1β and IL-18. In addition, depolarization of the mitochondrial membrane associated with iron-mediated oligomerization of Tom20 or other cell death signals leads to the release of cytochrome c and ultimately results in activation of caspase-3. Active caspase-3 can mediate cleavage of the N-terminal domains of gasdermin E (GSDME) that form pores, similarly to the N-terminal fragments of GSDMD.

A classical pathway of pyroptosis is triggered when cells activate inflammasomes, which are cytoplasmic multi-protein platforms that control maturation and secretion of cytokines. Inflammasomes are composed of the nucleotide-binding oligomerization domain (NOD)-like receptor (NLR) family: NLRP1, NLRP3, and NLRC4, absent in melanoma 2 (AIM2), and pyrin proteins through the action of pathogen-associated molecular patterns (PAMPs), DAMPs and homeostasis-altering molecular processes (HAMPs), which are diversely involved under different conditions and stimuli [213]. Inflammasomes activate caspase-1, which promotes both the maturation and secretion of pro-inflammatory cytokines, and cleaves GSDMD (Figure 5). Notably, while NLRP3-dependent activation of caspase-1 requires the contribution of apoptosis-associated speck-like (ASC) protein containing a caspase recruitment domain (CARD), NLRP1 activates caspase-1 in an ASC-independent manner due to the presence of a C-terminal CARD domain that facilitates direct association with caspase-1 and its activation via CARD–CARD interaction [214]. GSDMD has a highly conserved site between C-terminal (auto-inhibitory) and N-terminal domains recognized and cleaved by caspase-1, -4, -5, and -11. Following cleavage by caspases, the 30 kDa N-terminal (cNT) fragment of GSDMD (GSDMD-cNT) is released and binds to the components of the lipid bilayer, including phosphatidic acid, phosphatidylinositol and phosphatidylserine that results in pore formation to execute cell swelling and rupture [211,215]. In addition, caspase-1-processed IL-1β and IL-18 are released through pores formed by GSDMD-cNT (Figure 5) [214].

It has also been demonstrated that a non-classical pyroptosis can be triggered in response to activation of caspase-4, -5, and -11, which can produce a similar cleavage product of GSDMD followed by channel formation in the cellular membrane (Figure 5) [215]. Although caspase-4, -5, and -11 can interact with and activate caspase-1 in the presence of NLRP3 and ASC, caspase-1 is dispensable for cleavage of GSDMD in a non-classical pyroptosis [216]. More recently, it has been also demonstrated that caspase-3, involved in apoptosis, can cleave GSDME after Asp^270^, and generate an N-terminal fragment that mediates pyroptosis in a fashion similar to GSDMD-cNT-mediated cell death [217]. This was independently confirmed by others, and caspase-3-dependent cleavage of GSDME has been implicated as a mechanism underlying cancer cell response to several chemotherapeutics (Figure 5) [218].

### 5.2. Pyroptosis in Melanoma

As GSDME is expressed in the majority of melanomas [218], it may be an opportunity for exploiting pyroptosis as an alternative anti-melanoma therapeutic strategy. The abundance of GSDME can determine whether cells undergo either apoptosis or pyroptosis. Cell lines with high expression of *DFNA5* were committed to pyroptosis in response to different chemotherapeutics, while *DFNA5*-null cancer cells underwent apoptosis upon treatment with these drugs [218]. It has been shown that GSDME mediated doxorubicin-induced pyroptosis in melanoma cells [219]. Pyroptosis was further enhanced in cells exposed to doxorubicin when autophagy was inhibited, while the crosstalk between these two modalities of cell death was dictated by eukaryotic elongation factor-2 kinase (eEF-2K) [219]. In turn, etoposide-resistant melanoma cells expressed *DFNA5* at low level, but upregulation of *DFNA5* expression enhanced sensitivity of melanoma cells to etoposide [220]. It has also been found that pyroptosis was a way of oncolytic activity of herpes simplex virus type 2 (HSV-2) mutant ΔPK in a number of melanoma cell lines. HSV-2 mutant ΔPK activated calpain and caspase-3 and -7, and involved TNF-α, which activated NLRP3- and caspase-1-dependent pyroptosis [221]. More recently, it has been demonstrated that a combination of BRAFi and MEKi induced pyroptosis in melanoma cells. Following combined treatment, cleavage of GSDME, and increased expression and release of immunostimulatory molecules such as HMGB1 were reported. As a consequence, enhanced expression of major histocompatibility complex (MHC) class II on tumor-resident DCs was followed by tumor infiltration with T cells. Mechanistically, gasdermin E was indispensable for melanoma cell response as GSDME deficiency resulted in more frequent tumor re-growth when administration of drugs was ceased. Notably, BRAFi- and MEKi-resistant cells were still sensitive to pyroptosis-inducing compounds [222].

Pyroptosis can also be induced in melanoma by ROS that links pyroptosis and other cell death modalities. It has been demonstrated that iron-induced ROS signaling caused the oxidation and oligomerization of the subunit of mitochondrial import receptor, translocase of outer mitochondrial membrane (Tom20). Activated Tom20 facilitated recruitment of BAX to release cytochrome c and activate caspase-3, which eventually cleaved GSDME and induced pyroptosis (Figure 5) [223]. To validate the role of pyroptosis in melanoma, A375 cells either GSDME^wt^ or GSDME-deficient were separately injected subcutaneously into nude mice. It has been demonstrated that treatment of GSDME^wt^ mice with iron dextran promoted cleavage of GSDME and significantly decreased the tumor growth. In contrast, iron supplementation was ineffective in the reduction of tumor weight in GSDME-knockdown mice [223]. Thus, it has been suggested that supplementation of iron can maximize the anti-tumor effect of drugs that induce ROS to inhibit growth of melanoma tumor via GSDME-dependent pyroptosis [223].

Several studies have investigated the role of inflammasome components in melanoma. In general, RNA sequencing data of 458 melanoma specimens have revealed that low expression of inflammasome-related mediators are predictive of poor prognosis in melanoma patients [224]. It has been demonstrated that both primary and metastatic melanoma cells possessed activated inflammasomes that was associated with co-expression of *ASC* and *NLRP3*, whereas self-dependence of melanoma cells on IL-1β signaling was shown to increase with the stage of disease [225]. A melanoma-specific role of other inflammasome-related protein NLRP1 has also been envisaged [226]. It has been shown that NLRP1 exhibited a dual role as it promoted melanoma growth by activating inflammasome and suppressing apoptosis. Inhibition of apoptosis by NLRP1 was evidenced by immunoprecipitation that revealed interaction between CARD-containing NLRP1 with similar domains in caspase-2 and caspase-9. In addition, NLRP1 was mainly confined in the cytosol of melanoma cells, suggesting that cellular localization of NLRP1 might also promote its interaction with caspases. Downregulation of NLRP1 promoted apoptosis in both primary and metastatic melanoma. However, further study revealed an additional function of NLRP1 assessed exclusively in metastatic melanoma cells. Mechanistic studies showed that cells employed NLRP1-dependent inflammasomes, and NLRP1 knockdown was associated with reduced activity of caspase-1, diminished secretion of IL-1β, and decreased activity of NF-κB in metastatic melanoma cells [226]. Interestingly, also other inflammasome-related proteins have been shown to exert melanoma stage-dependent function. Expression of *ASC* was downregulated in metastatic melanoma compared with primary tumor specimens. In contrast, elevated level of ASC inhibited NF-κB activity and affected processing of IL-1β in primary melanoma cells [227]. This suggests that the function of ASC switches from anti-cancer activity in primary melanoma cells to pro-tumorigenic in metastatic melanoma. Other members of the gasdermin family of proteins have also been detected in melanoma as shown for *GSDMC* that was not expressed in the normal epidermis, but its elevated level in melanoma cells correlated with their metastatic potential [228].

## 6. Conclusions and Future Directions

It is evident that melanoma cells utilize multiple mechanisms to counteract cell death-inducing signals, and the intersections between different signaling pathways involved in the regulation of cell survival and death are presumably a reason for a failure in melanoma response to potentially pro-death triggers. The landscape of regulated cell death modalities is still enlarging, and the role of non-apoptotic cell death signaling pathways have been given increasing attention in melanoma. This is further exemplified by parthanatos, which is a modality of cell death initiated by hyperactivation of PARP1 that results in depletion of both NAD+ and ATP, and accumulation of poly(ADP-ribosyl)ated proteins in the mitochondria [5,229,230]. This is followed by the release of apoptosis-inducing factor (AIF) into the cytosol and its subsequent translocation to the nucleus, where it mediates DNA fragmentation in cooperation with macrophage migration inhibitory factor (MIF) [231,232], but without contribution of caspases and endonuclease G (ENDOG) [233]. Parthanatos has been indicated as a cell death modality in melanoma cells that acquired resistance to BRAFi and/or MEKi, and it was preferentially induced upon drug withdrawal in cell lines characterized with high levels of active ERK1/2 rebound in response to drug cessation [234].

The reports discussed in the present review largely suggest that melanoma cell vulnerability to induction of cell death markedly depends on the microenvironment insults, including anti-cancer drugs, and can extensively evolve during acquisition of drug resistance. This implicates non-apoptotic types of regulated cell death to be considered as alternative therapeutic targets when induction of apoptosis is impaired. However, this also raises a question how uniform the sensitivity of melanoma cells to induction of particular type of cell death is, considering large patient-to-patient variability of drug resistance mechanisms, which can be both genetic and non-genetic [124,235,236]. In this respect, searching of molecular markers that correlate the phenotype of melanoma cells with vulnerability to different death-inducing triggers, and identifying transcriptional regulators responsible for controlling acquisition of this phenotype should be the directions of current research.

## Figures and Tables

**Table 1:** The comparison of major morphological and biochemical features and markers of apoptosis and non-apoptotic cell death modalities [12,13,14,15].

	Apoptosis	Autophagy	Necroptosis	Ferroptosis	Pyroptosis
morphology	membrane blebbing	formation of intracellular vesicles	cell swelling	shrunken mitochondria with reduced crista	cell swelling; pore formation
DNA fragmentation	+	-	+ (random)	-	+
caspase activation	+	-	-	-	+
positive staining with Annexin-V	+	-	+	+	+
inflammation	-	-	+	+	+
other typical features	PARP cleavage	LC3-I to LC3-II conversion; p62/SQSTM1 degradation	RIPK3/MLKL activation; drop in ATP level	iron accumulation and lipid peroxidation	osmotic lysis of cell

## Abbreviations

*ATG*s	autophagy-related genes
BCL-2	B-cell leukemia/lymphoma 2
BNIP3	BCL-2/adenovirus E1B 19 kDa protein-interacting protein 3
BRAF	B-Raf proto-oncogene serine/threonine kinase
BRAFi	inhibitors of mutated BRAF
CDR1as	cerebellar degeneration-related 1 antisense
DAMPs	damage-associated molecular patterns
DC	dendritic cell
GPX4	glutathione peroxidase 4
GSDM	gasdermin
GSH	glutathione (reduced)
HMGB1	high mobility group box 1
HSP90	heat shock protein 90
IFN	interferon
IL-1β	interleukin-1β
LC3	microtubule-associated protein light chain 3
MEKi	inhibitors of MEK1/2
MITF	microphthalmia-associated transcription factor
MLKL	mixed lineage kinase domain-like
NF-κB	nuclear factor-kappa B
NGFR	nerve growth factor receptor
NK	natural killer
NRF-2	nuclear factor erythroid 2-related factor 2
PD-L1	programmed death-ligand 1
OXPHOS	oxidative phosphorylation
RCD	regulated cell death
RIPK	receptor-interacting protein kinase
ROS	reactive oxygen species
SQSTM1/p62	sequestosome 1
TNF-α	tumor necrosis factor alpha
UV	ultraviolet
WNT	Wingless-type
